# Inhalation injury is associated with long-term employment outcomes in the burn population: Findings from a cross-sectional examination of the Burn Model System National Database

**DOI:** 10.1371/journal.pone.0239556

**Published:** 2020-09-23

**Authors:** Olivia R. Stockly, Audrey E. Wolfe, Gretchen J. Carrougher, Barclay T. Stewart, Nicole S. Gibran, Steven E. Wolf, Kara McMullen, Alyssa M. Bamer, Karen Kowalske, William G. Cioffi, Ross Zafonte, Jeffrey C. Schneider, Colleen M. Ryan

**Affiliations:** 1 Department of Physical Medicine and Rehabilitation, Spaulding Rehabilitation Hospital, Charlestown, MA, United States of America; 2 Department of Surgery, University of Washington, Seattle, WA, United States of America; 3 Harborview Injury Prevention and Research Center, Seattle, WA, United States of America; 4 Department of Surgery, University of Texas Medical Branch, Galveston, TX, United States of America; 5 Department of Rehabilitation Medicine, University of Washington, Seattle, WA, United States of America; 6 Department of Physical Medicine and Rehabilitation, University of Texas Southwestern Medical Center, Dallas, TX, United States of America; 7 Department of Surgery, Brown University, Rhode Island Hospital, Providence, RI, United States of America; 8 Department of Surgery, Massachusetts General Hospital, Harvard Medical School, Boston, MA, United States of America; 9 Shriners Hospitals for Children—Boston, Boston, MA, United States of America; US Army Institute of Surgical Research, UNITED STATES

## Abstract

**Introduction:**

Inhalation injuries carry significant acute care burden including prolonged ventilator days and length of stay. However, few studies have examined post-acute outcomes of inhalation injury survivors. This study compares the long-term outcomes of burn survivors with and without inhalation injury.

**Methods:**

Data collected by the Burn Model System National Database from 1993 to 2019 were analyzed. Demographic and clinical characteristics for adult burn survivors with and without inhalation injury were examined. Outcomes included employment status, Short Form-12/Veterans Rand-12 Physical Composite Score (SF-12/VR-12 PCS), Short Form-12/Veterans Rand-12 Mental Composite Score (SF-12/VR-12 MCS), and Satisfaction With Life Scale (SWLS) at 24 months post-injury. Regression models were used to assess the impacts of sociodemographic and clinical covariates on long-term outcome measures. All models controlled for demographic and clinical characteristics.

**Results:**

Data from 1,871 individuals were analyzed (208 with inhalation injury; 1,663 without inhalation injury). The inhalation injury population had a median age of 40.1 years, 68.8% were male, and 69% were White, non-Hispanic. Individuals that sustained an inhalation injury had larger burn size, more operations, and longer lengths of hospital stay (p<0.001). Individuals with inhalation injury were less likely to be employed at 24 months post-injury compared to survivors without inhalation injury (OR = 0.63, p = 0.028). There were no significant differences in PCS, MCS, or SWLS scores between groups in adjusted regression analyses.

**Conclusions:**

Burn survivors with inhalation injury were significantly less likely to be employed at 24 months post-injury compared to survivors without inhalation injury. However, other health-related quality of life outcomes were similar between groups. This study suggests distinct long-term outcomes in adult burn survivors with inhalation injury which may inform future resource allocation and treatment paradigms.

## Introduction

There is growing interest in the long-term outcomes of those with inhalation injury. Approximately 1,400 patients per year with recorded smoke inhalation injury were entered into the American Burn Association’s National Burn Registry between 2008 and 2017. Burn-related mortality rates remain low at 1.5% in the United states, though inhalation injury increases the mortality rate by 17.5% [1]. The National Academies of Sciences, Engineering, and Medicine recently held a multidisciplinary workshop to examine the implications of the California wildfires on health, communities, and preparedness [2]. The workshop identified the lack of long-term health data of large populations with smoke exposure to a single fire and possibly multiple fires over a lifetime [2].

Studies examining long-term outcomes of inhalation injury are limited by small sample sizes or animal models [3–6]. Default proxy for diagnosis of inhalation injury is through history and physical exam, as only a few diagnostic procedures have been proposed and validated [7–12]. Smoke inhalation is difficult to study given that each exposure is different and components of the smoke vary at each fire and at different locations within the same fire [10]. Although rates of mortality following inhalation injury have improved in the past few decades, it remains unclear whether this is due to improvements in the diagnosis and treatment of inhalation injury or critical illness more generally [7, 13, 14]. Inhalation injury is closely tied to increases in hospital-acquired infections, most frequently ventilator-associated pneumonia, a significant contributor to morbidity and mortality [15, 16]. Efforts to disentangle the inhalation injury itself from the long-term outcomes related to critical illness are limited by the available data [17]. A study of survivors of the King’s Cross fire demonstrated an association between inhalation injury and long-term damage to the small airways, though it was limited by a small sample size and did not examine any psychosocial or physical outcomes beyond respiratory disability [18]. However, reports have not consistently demonstrated that inhalation injury causes lasting morbidity. For example, Witt et. al reported that inhalation injury increased the odds of long-term mortality and pulmonary morbidity post-discharge. Conversely, Bourbeau et. al described that exposure to smoke inhalation from a fire does not necessarily result in long-term respiratory effects up to 45 months post-injury [5, 19]. Further, one study did not find inhalation injury had any effect on quality of life, though was limited to the pediatric population [6]. Palmieri described a lack of data on long-term outcomes following inhalation injury and outlined potential avenues for future study [20]. The acute sequelae of inhalation injury and the associated morbidity and mortality have been studied, yet the long-term outcomes of inhalation injury remain elusive, and require examination.

The physical and psychosocial outcomes of burn survivors with inhalation injury remain underexplored. The Burn Model System National Database presents an opportunity to compare long-term outcomes of those with inhalation injury to those without. A better understanding of the long-term outcomes of this population will inform future efforts to provide anticipatory guidance to patients and allocate recovery resources to health systems that care for people living with inhalation injury. This study aims to examine a very gross measure of smoke inhalation and long-term effects. Authors anticipate outcomes to be related to anoxic events and decreased lung function or lung capacity from scarring. Therefore, impairments are expected in endurance, fine cognitive function, and neurological sequelae.

## Methods

### Database

A retrospective study was conducted using data from the Burn Model System (BMS) National Database. The BMS National Database was created in 1993 as a means of exploring the long-term physical and social outcomes of burn survivors and is funded by the National Institute on Disability, Independent Living, and Rehabilitation Research. Six burn centers have contributed to the database since its creation [21]. The BMS National Database is a publicly accessible database and information on accessing the dataset can be found on http://www.burndata.washington.edu. Authors has no special access privileges in accessing this database. Data is collected at time of discharge from the acute care hospital and at 24±6 months post-injury. Written informed consent is obtained from all participants. Institutional Review Boards from Partners Healthcare, University of Texas Medical Branch, University of Washington, Parkland Health & Hospital System, University of Colorado, and Johns Hopkins University approved the study. Data was analyzed anonymously. Participants who were burned between 1993 and 2019, aged 18 years or older at time of injury, and alive at discharge were included in this study. For the purposes of analysis participants were divided into two groups using the inhalation injury variable (coded as “yes/no inhalation injury”), those with and without inhalation injury. The inhalation injury variable in the BMS Database is based on the clinical judgement of the attending physician and is determined through documentation in the medical record. The database does not have a standardized method for determining inhalation injury across all data collection sites. International Classification of Diseases (ICD) codes are not used to identify inhalation injury in the Database. ICD codes associated with inhalation injury have evolved over time and exhibit low specificity for the diagnosis [22]. Current criteria for enrollment in the BMS Database are those who require autografting surgery for wound closure and meet one of the following criteria:

1. 0–64 years of age with a burn injury ≥20% total body surface area (TBSA) **OR**
2. ≥65 years of age with a burn injury ≥10% TBSA **OR**
3. Any age with a burn injury to their face/neck, hands, or feet **OR**
4. Any age with a high-voltage electrical burn injury

The BMS National Database inclusion criteria have been modified since the database’s creation. Further details regarding data collection, inclusion criteria, and data sites was previously published and can be found at http://burndata.washington.edu [21]. The BMS National Database is a centralized database that utilizes REDCap electronic data capture tools and is housed at the BMS National Data and Statistical Center at the University of Washington [23].

### Demographic and clinical characteristics

Demographic data included age, gender, race/ethnicity, highest education level (associates degree or higher; high school or less), pre-injury employment status (working; not working or retired), circumstances of injury (employment related injury; non-employment related injury), and etiology of injury (fire/flame; other). Clinical data included burn and graft size (total body surface area (TBSA) burned and grafted), length of stay, number of days in the intensive care unit (ICU), number of trips to the operating room, and number of days on ventilator. Demographic and clinical characteristics were collected through self-report and medical record abstraction at time of discharge from acute care.

### Outcome measures

The following patient reported outcome measures were used to evaluate employment status, physical health, mental health, and life satisfaction at 24 months post-injury.

#### 1. Employment status

Employment status is collapsed into two categories: working and not working. Not working included those looking for work, those not looking for work, homemaker/caregivers, volunteers, and those retired.

#### 2. The Short Form-12 version 2 and Veterans Rand-12 Health Survey

The Short Form-12 (SF-12) and Veterans Rand-12 Health Survey (VR-12) are standardized, clinically validated evaluations of general health and ability that are often used in research. The SF-12 was collected from 1997–2015 and the VR-12 was collected from 2015–2019 in the BMS Database. They were created as shortened versions of the Short Form Health Survey and the Veterans Rand 36 Item Health Survey, respectively [24, 25]. Both the SF-12 and VR-12 are comprised of two sub-scores: the Physical Health Composite Scale (PCS) and the Mental Health Composite Scale (MCS). SF-12 and VR-12 scores are interconvertible [26]. PCS and MCS scores are based on the United States population and are standardized through t-score transformation with a mean of 50, standard deviation of 10, and a maximum score of 100 [27]. Lower PCS and MCS scores are associated with poorer physical and mental health, respectively.

#### 3. Satisfaction with Life Scale

The Satisfaction with Life Scale (SWLS) is a validated scale in burn, traumatic brain injury, and spinal cord injury populations comprised of 5 items addressing life satisfaction and well-being [28, 29]. Each of the 5 items are scored on a 1–7 Likert scale, with a maximum score of 35; higher scores are associated with increased satisfaction with life.

### Data analysis

Demographic and clinical characteristics of those with and without inhalation injury were compared using Chi-square tests of association and Wilcoxon-Mann Whitney rank tests for categorical and continuous variables, respectively. The Wilcoxon-Mann Whitney test was used due to non-normality of multiple variables. Differences between groups for each of the four outcome variables (employment status, PCS, MCS, and SWLS) were similarly assessed using Chi-square and Wilcoxon-Mann Whitney rank tests followed by multivariate regression modeling. A logistic regression model was used to assess the association between inhalation injury and employment status at 24 months post-injury. Linear regression models were used to examine the association between inhalation injury and PCS, MCS, and SWLS. All regression models controlled for age, gender, race/ethnicity, burn size, pre-injury employment status, etiology of injury, employment-related injury, ventilator days, length of hospital stay, and number of trips to the operating room. The ICU days variable was only collected through 2015 and therefore was not included in regression analyses due to missing data. Variables were included in the models regardless of significance. Robust standard errors were calculated for all regression models. A p-value less than 0.05 was considered statistically significant for all regression analyses. A Bonferroni adjustment of significance was used on tests of group differences (Table 1) due to multiple comparisons, with a p-value less than 0.003 considered statistically significant. Model assumptions examined multicollinearity, linearity, normality, homoscedasticity, and outlying or high leverage points. Due to the non-linearity of age and burn size, both variables were converted to categorical variables (Age: 18–29 (reference category), 30–49, 50–59, 60+; burn size: 0–30% (reference category) 30–59%, 60–99%). Additionally, individuals with length of stay greater than 100 days were recoded to 100 due to problems with linearity outside 100 days.

**Table 1:** Demographic and clinical characteristics of the study population*.

Variable	Inhalation Injury (n = 208)	No Inhalation Injury (n = 1663)	p-value
Age, median years (IQR)	40.1 (31.8–50.5)	43.2 (32.0–54.4)	0.06
Male, % (n)	68.8 (143)	73.5 (1,222)	0.15
Race/ethnicity, % (n)			0.30
White, non-Hispanic	69 (144)	72 (1,194)	
Black, non-Hispanic	10 (20)	12 (194	
Hispanic	13 (27)	11 (182)	
Other^+^	8 (17)	6 (93)	
Working pre-injury, % (n)	63 (131)	67 (1,122)	<0.001
Highest education level, % (n)			0.001
Less than high school	11 (10)	3(21)	
High school diploma/GED	65 (59)	69 (516)	
Associate degree	10 (9)	14 (102)	
Bachelor’s degree or higher	14 (13)	15 (114)	
Fire/flame injury, % (n)	88 (184)	57 (942)	<0.001
Employment related injury, % (n)	19 (40)	30 (493)	<0.002
Burn size (% TBSA), median (IQR)	31.3 (17–50)	13.0 (5.5–25)	<0.001
Graft size, (%TBSA), median (IQR) [n]^#^	18.7 (6–34) [195]	5 (1.5–12) [1,450]	<0.001
Length of hospital stay, median days (IQR)	47 (25–75)	20 (13–32)	<0.001
ICU admission, % (n) ^β^	95.2 (99)	50.9 (475)	<0.001
ICU stay, median days (IQR) [n]^β^	21 (7.5–39) [104]	1 (0–8) [933]	<0.001
Trips to the operating room, median (IQR)	3 (1–7)	1 (1–2)	<0.001
Ventilator use, median days (IQR)	10 (2–26)	0 (0–0)	<0.001
Return to work, median days (IQR) [n]	180 (101–406) [63]	94 (55–380) [596]	<0.001

TBSA = Total Body Surface Area.

GED = General Education Development.

ICU = Intensive Care Unit.

IQR = Interquartile Range.

*Chi-square tests of association and Wilcoxon-Mann Whitney tests for categorical and continuous variables were used, respectively.

^+^Other race includes Asian; American Indian/Alaskan native; Native Hawaiian or Other Pacific Islander; more than one race; and other race.

^#^Median (IQR) variables note the size of the group in the case of incomplete data.

^β^The ICU days variable was not collected after 2015.

### Sensitivity analysis

Given the presence of missing data at follow up, a sensitivity analysis was used to assess for selection bias through examination of differences in demographic and clinical characteristics between those with outcome data and those without outcome data at 24 months post-injury.

## Results

### Demographic and clinical characteristics

A total of 1,871 individuals were included in the study (208 with inhalation injury and 1,663 without inhalation injury). Those with inhalation injury had larger burn size (31.3 vs. 13.0% TBSA; p<0.001), larger graft size (18.7 vs. 5% TBSA; p<0.001), were less likely to be working at time of injury (63% vs. 67%; p<0.001), more likely to have sustained a fire/flame injury (88% vs. 57%; p<0.001), and were less likely to have sustained a work-related injury (19% vs. 30%; p<0.002) than those without inhalation injury. Those with inhalation injury also had longer acute care lengths of stay (47 vs. 20 days; p<0.001), more ICU days (21 vs. 1 days; p<0.001), more trips to the operating room (3 vs. 1; p<0.001), more days on the ventilator (10 vs. 0 days; p<0.001), and took longer to return to work (180 vs. 94 days; p<0.001) compared to those without inhalation injury. Demographic and clinical characteristics are summarized in Table 1.

### Comparison of outcome measures

Comparison of the outcome measures demonstrated that those with inhalation injury were less likely to be employed (32% vs. 56%; p<0.001), have worse physical health (PCS: 42.8±12.0 vs. 46.9±10.5; p = 0.0001) and worse satisfaction with life (SWLS: 19.4±9.0 vs. 22.2±8.4; p = 0.002) compared to those without inhalation injury at 24 months post-injury. Mental health (MCS) was not significantly different between the two groups (p = 0.23) (Table 2).

**Table 2:** Comparison of outcome measures between those with and without inhalation injury at 24 months post-injury*.

Outcome	Inhalation Injury	No Inhalation Injury	p-value
Employment status, % (n)			<0.001
Working	32 (64)	56 (910)	
Not working	57 (113)	21 (507)	
Retired	11 (21)	12 (202)	
PCS, mean (SD)	42.8 (12.0)	46.9 (10.5)	0.0001
MCS, mean (SD)	47.0 (12.8)	48.6 (12.2)	0.23
SWLS, mean (SD)	19.4 (9.0)	22.2 (8.4)	0.002

PCS = Physical Component Score.

MCS = Mental Component Score.

SWLS = Satisfaction With Life Score.

* Chi-square tests of association and Wilcoxon-Mann Whitney tests for categorical and continuous variables were used, respectively.

### Regression analyses

In logistic regression analyses, burn survivors with inhalation injury were 0.63 times as likely to be employed at 24 months post-injury compared to those without inhalation injury (OR = 0.63, p = 0.028), controlling for demographic and clinical variables (Table 3). Another way to state this result is that burn survivors without inhalation injury were 1.58 times as likely to be working at 24 months compared to those with inhalation injury. There was no association between inhalation injury and PCS scores (p = 0.350), MCS scores (p = 0.325) or SWLS scores (p = 0.119), in adjusted linear regression models (Tables 4–6).

**Table 3:** Logistic regression analysis examining the association between employment status at 24 months post-injury and inhalation injury.

Number of observations = 1,817					
Wald chi^2^ (16) = 475.95					
Pseudo R^2^ = 0.2997					
Variable	OR	Robust SE	z	95% CI	
Inhalation injury*	0.63	0.13	-2.20	0.42	0.95
Age					
30–49 years*	0.60	0.10	-3.01	0.43	0.84
50–59 years*	0.41	0.08	-4.56	0.28	0.60
60+ years *	0.18	0.04	-7.86	0.12	0.27
Burn size (TBSA)					
30–59%	0.82	0.16	-1.05	0.56	1.19
60–99%	1.06	0.41	0.15	0.50	2.25
Race/ethnicity					
Black or African American*	0.48	0.09	-3.76	0.33	0.70
Hispanic	0.74	0.14	-1.61	0.51	1.07
Other	0.99	0.24	-0.05	0.61	1.59
Female gender*	0.63	0.09	-3.29	0.47	0.83
Employed at time of burn*	11.78	1.82	15.97	8.70	15.94
Non-fire/flame injury	0.90	0.12	-0.78	0.70	1.17
Ventilator days	0.99	0.01	-0.85	0.98	1.01
Length of acute hospital stay (days)*	0.98	0.005	-4.28	0.97	0.99
Trips to the operating room	0.92	0.04	-1.82	0.85	1.01
Employment-related injury	0.74	0.11	-1.99	0.55	1.00

*indicates p<0.05.

TBSA = Total body surface area.

OR = Odds ratio.

SE = Standard error.

CI = Confidence interval.

Age reference group: 18–29 years; TBSA reference group: 0–29%.

**Table 4:** Linear regression analysis examining the association between standardized PCS score and inhalation injury at 24 months.

Number of observations = 1,178					
F(16, 1161) = 12.39					
R^2^ = 0.1595					
Variable	Coef.	Robust SE	t	95% CI	
Inhalation injury	-1.05	1.12	-0.94	-3.24	1.15
Age					
30–49 years*	-3.35	0.72	-4.64	-4.77	-1.93
50–59 years*	-5.57	0.89	-6.23	-7.33	-3.82
60+ years*	-4.72	1.01	-4.66	-6.70	-2.73
Burn size (TBSA)					
30–59%	0.56	0.91	0.61	-1.22	2.33
60–99%	0.84	1.82	0.46	-2.73	4.41
Race/ethnicity					
Black or African American*	-3.13	0.87	-3.59	-4.84	-1.42
Hispanic	0.20	0.84	0.24	-1.46	1.85
Other	1.27	1.49	0.86	-1.64	4.19
Female gender	-0.49	0.68	-0.72	-1.83	0.85
Employed at time of burn*	3.99	0.76	5.27	2.51	5.48
Non-fire/flame injury	-0.87	0.66	-1.31	-2.16	0.43
Ventilator days	-0.02	0.03	-0.57	-0.08	0.04
Length of acute hospital stay (days)*	-0.13	0.03	-5.12	-0.19	-0.08
Trips to the operating room	0.03	0.20	0.14	-0.37	0.42
Employment-related injury*	-3.52	0.74	-4.75	-4.988	-2.07

PCS = Physical Component Summary of the SF-12.

*indicates p<0.05.

TBSA = Total body surface area.

Coef = Coefficient.

SE = Standard error.

CI = Confidence interval.

Age reference group: 18–29 years; TBSA reference group: 0–29%.

**Table 5:** Linear regression analysis examining the association between standardized MCS score and inhalation injury at 24 months.

Number of observations = 1,178					
F(16, 1161) = 4.80					
R^2^ = 0.0635					
Variable	Coef.	Robust SE	t	95% CI	
Inhalation injury	-1.26	1.28	-0.98	-3.76	1.25
Age					
30–49 years	-1.80	0.93	-1.94	-3.62	0.02
50–59 years	-0.95	1.05	-0.90	-3.02	1.11
60+ years*	3.30	1.25	2.64	0.84	5.75
Burn size (TBSA)					
30–59%*	-2.45	1.07	-2.30	-4.54	-0.36
60–99%	0.30	2.09	0.14	-3.81	4.40
Race/ethnicity					
Black or African American*	-2.81	1.08	-2.61	-4.92	-0.69
Hispanic	1.19	1.11	1.07	-0.99	3.36
Other	0.28	1.52	0.18	-2.70	3.26
Female gender*	-3.50	0.84	-4.17	-5.15	-1.86
Employed at time of burn*	2.95	0.92	3.22	1.15	4.75
Non-fire/flame injury	-0.12	0.80	-0.14	-1.69	1.46
Ventilator days	0.03	0.04	0.92	-0.04	0.10
Length of acute hospital stay (days)	-0.03	0.03	-0.87	-0.08	0.03
Trips to the operating room	0.12	0.21	0.56	-0.30	0.53
Employment-related injury	-1.70	0.87	-1.96	-3.40	0.00

MCS = Mental Component Summary of the SF-12.

*indicates p<0.05.

TBSA = Total body surface area.

Coef = Coefficient.

SE = Standard error.

CI = Confidence interval.

Age reference group: 18–29 years; TBSA reference group: 0–29%.

**Table 6:** Linear regression analysis examining the association between standardized SWLS score and inhalation injury at 24 months.

Number of observations = 1,086					
F(16, 1069) = 6.46					
R^2^ = 0.0800					
Variable	Coef.	Robust SE	t	95% CI	
Inhalation injury	-1.49	0.95	-1.56	-3.36	0.38
Age					
30–49 years*	-1.48	0.66	-2.25	-2.78	-0.19
50–59 years*	-2.07	0.80	-2.60	-2.63	-0.51
60+ years	1.69	0.91	1.86	-0.10	3.48
Burn size (TBSA)					
30–59%	-0.71	0.82	-0.86	-2.32	0.90
60–99%	1.17	1.62	0.72	-2.02	4.35
Race/ethnicity					
Black or African American*	-1.99	0.71	-2.81	-3.38	-0.60
Hispanic*	3.22	0.76	4.26	1.73	4.70
Other	0.53	1.33	0.40	-2.07	3.14
Female gender	-0.38	0.60	-0.63	-1.55	0.80
Employed at time of burn*	2.07	0.64	3.24	0.82	3.32
Non-fire/flame injury	-0.11	0.56	-0.20	-1.21	0.98
Ventilator days	-0.005	0.03	-0.18	-0.06	0.05
Length of acute hospital stay (days)*	-0.04	0.02	-2.18	-0.08	-0.004
Trips to the operating room	-0.02	0.13	-0.13	-0.28	0.25
Employment-related injury	-1.00	0.63	-1.59	-2.24	0.24

SWLS = Satisfaction With Life Score.

*indicates p<0.05.

TBSA = Total body surface area.

Coef = Coefficient.

SE = Standard error.

CI = Confidence interval.

Age reference group: 18–29 years; TBSA reference group: 0–29%.

### Sensitivity analysis

**Fig 1:**
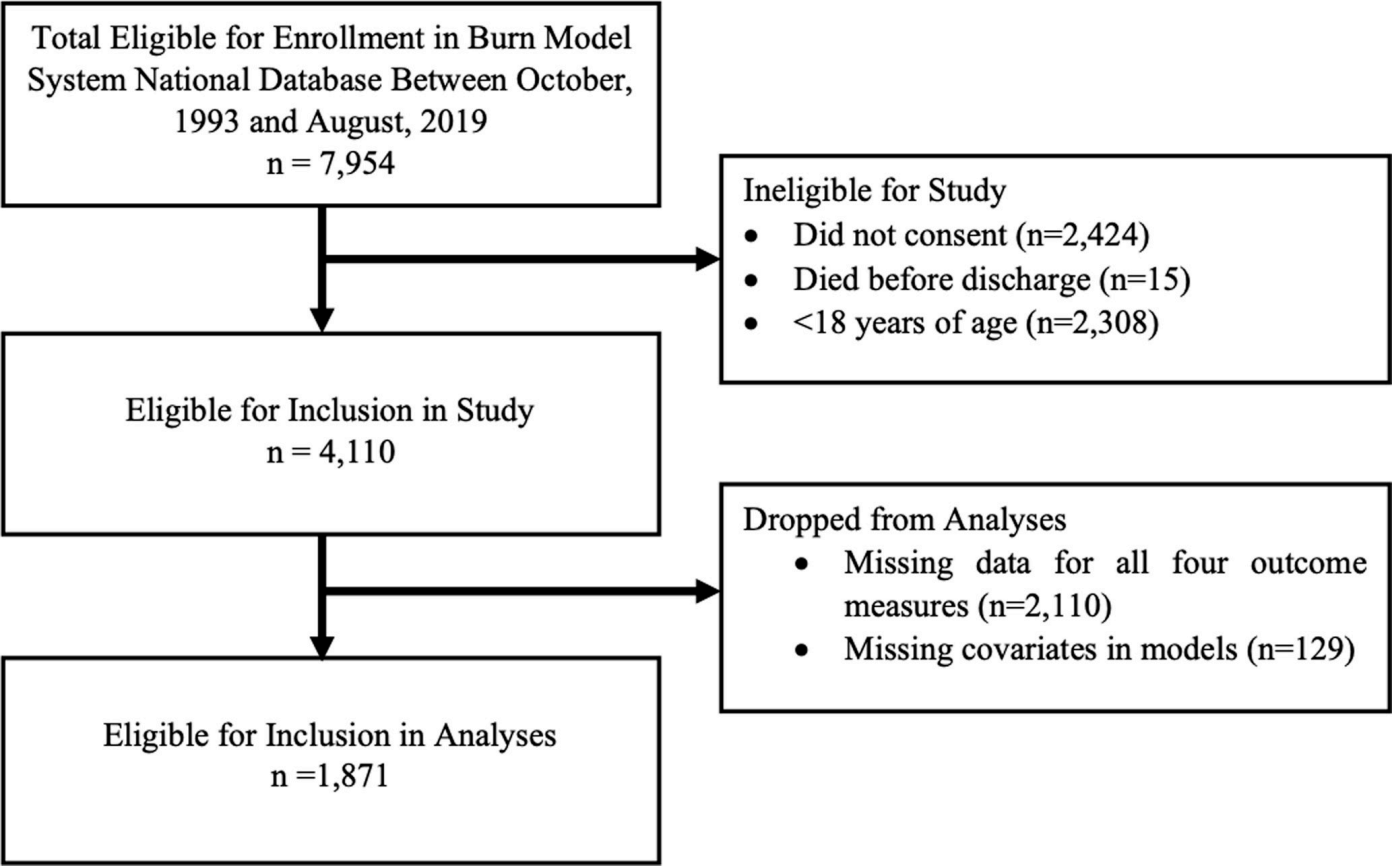
Flowchart of inclusion and exclusion criteria.

This study had 4,110 eligible participants with 2,110 participants missing all four outcome measures and 129 missing model covariates. Further details regarding inclusion and exclusion criteria can be found in Fig 1. Participants with follow-up data at 24 months post-injury were more likely to be older (p<0.001), White, non-Hispanic (p<0.001), and have a larger burn size (p<0.001). Additionally, those with longer lengths of acute stay (p<0.001) and more days on a ventilator (p = 0.011) were more likely to have follow-up data. There were no significant differences in follow-up rates by gender or etiology of injury. There was no difference in follow-up rate between those with inhalation injury and those without inhalation injury.

## Discussion

The long-term implications of inhalation injury have been largely undocumented, with few studies examining markers of recovery or quality of life outcomes measures [6, 20]. This study compared long-term outcomes of burn survivors with and without inhalation injury using the Burn Model System National Database. Participants with inhalation injury were less likely to be working at 24 months post-injury compared to those without inhalation injury. However, measures of physical health, mental health, and satisfaction with life were not associated with inhalation injury at 24 months post-injury after adjustment for demographic and clinical characteristics.

Employment is an important marker of overall recovery for burn survivors, as it indicates social participation, community integration, and return to livelihood for many individuals [3, 30]. For survivors who were working pre-injury, employment can signify a return to pre-injury activity and capabilities. Further, nearly 30% of all burn injuries occur at the workplace [31]. Therefore, the ability to return to work can be an important milestone for survivors. Schneider et. al found that burn survivors who sustained their injuries at work were significantly less likely to have returned to work by one year post-injury compared to employed patients burned outside of work [3]. In the general population, unemployment is associated with increased morbidity and mortality rates; conversely, employment is associated with higher quality of life [32, 33]. Following burn injury, survivors’ return to work is often limited by barriers including pain, psychiatric issues, and neurologic problems [3]. Pre-injury employment and post-acute care setting have both been found to be predictors of post-burn employment [34, 35]. Although length of stay was controlled for in the analysis, longer hospital stay is a predictor of unemployment at 12 months post-injury [3]. Burn survivors that do return to work frequently require job modification and accommodation [36]. Lower rates of return to work following inhalation injury may be due to an inability to properly accommodate such injuries. Burn survivors report inadequate work accommodations, with employers neglecting cognitive and psychosocial impairments, symptoms frequently observed in acute respiratory distress syndrome patients [37, 38]. Even more, inhalation injury does not necessarily result in a physical limitation such as contractures or itching, and may be harder for survivors to qualify needed accommodations to employers or insurers. Long-term inhalation injury sequelae such as interstitial fibrosis and delayed onset of respiratory failure are rare outcomes, but pose as possible obstacles to returning to work following injury and necessitate further exploration [39, 40]. Lower rates of employment could be related to hidden biopsychosocial impacts of inhalation injury or incidences of hypoxia, mirroring the long-term effects of acute respiratory distress syndrome [38, 41].

Many of the long-term outcomes of inhalation injury are also observed in critical illness populations, though this study attempted to control for this in analyses. Inhalation injury carries significant acute critical illness burden, including prolonged ICU stays and numerous days on the ventilator [9, 42]. This study corroborated previous findings, with inhalation injury participants having longer lengths of stay, more ventilator days, and more trips to the operating room. Because of the close relationship between inhalation injury and general critical illness, it is difficult to identify the contribution of each of these components to the studied outcomes. Long-term acute respiratory distress syndrome outcomes depict prolonged anxiety, depression, and posttraumatic stress disorder symptoms; sepsis survivors report fatigue, physical impairment, difficulty returning to normal living, and cognitive impairment [43, 44]. Critical illness also carries high risk of mortality in the years after discharge [45, 46]. This study attempted to control for critical illness factors by including the following available BMS variables in regression models: ventilator days, length of hospital stay, number of trips to the operating room, and burn size. Due to the close relationship between inhalation injury and critical illness, a multicollinearity analysis was examined as part of model assumptions and no co-linearity was observed. ICU stay was not included in regression analyses due to the high percentage of missing data for this variable due to changes in data collection variables over time (78% missing). A multi-collinearity analysis was examined between inhalation injury and ICU stay and no collinearity was observed.

In this study measures of physical health, mental health, and satisfaction with life were not associated with inhalation injury in linear regressions. Despite this, both groups displayed below average scores compared to the United States population in PCS and MCS outcomes [27]. Though the comparison of outcome measures displayed significant differences in measures of physical health and satisfaction with life between groups, these comparisons did not control for important demographic and clinical factors. Long-term physical and psychological outcomes following burn injury have been studied in detail, with survivors experiencing itching, contractures, and fatigue, as well as depression, anxiety, and post-traumatic stress disorder following injury [47–49]. Though survivors experience sequelae for decades following injury, only recently have burn injuries begun to be recognized as a chronic condition [50, 51]. Long-term life satisfaction, an important marker of health-related quality of life in the burn population, is consistently lower among burn survivors compared to non-burned groups [52, 53]. Improvements in these domains may aid both inhalation and non-inhalation injury survivors in returning to work and their community [36].

### Limitations

There are several limitations of this study that should be addressed. First, the inclusion criteria for the BMS Database selects those with more severe injuries, limiting the generalizability of results. However the database has been shown to be representative of the national burn population [54]. Due to the BMS Database enrollment criteria requiring autografting for wound closure, the BMS Database is unable to address outcomes of individuals with inhalation injury without cutaneous burn. However, the strength of this database is the comparison group with similar injuries without inhalation injury. Further, the variable “inhalation injury” was used to define the study population, though there is no additional information regarding the method of diagnosis, extent of exposure, severity of inhalation injury, or associated hypoxia included in the database. This is reflective of a lack of consensus on how to diagnose and grade inhalation injury [12, 17]. Validated markers of illness severity are not available in the dataset, therefore use of a traditional calculator (e.g. APACHE II) is not possible in this study. The outcomes of this study are not immediately generalizable to other non-burn related inhalation injury such as wildfire smoke inhalation; however, given the limited data on long-term inhalation outcomes, this data on burn inhalation may be a useful initial step in improving our understanding on inhalation injury outcomes more globally. This study was not able to evaluate changes in employment status over time or more granular details regarding employment outcomes, such as need for work accommodations or part-time work [36]. Additionally, cognitive and direct behavioral metrics were limited to the variables collected in the BMS Database. Lastly, this study utilized patient reported outcome measures, which are validated means of examining long-term outcomes in the burn and non-burn population [55, 56].

## Conclusions

Burn survivors without inhalation injury were 1.58 times as likely to be working at 24 months post-injury compared to those with inhalation injury. Inhalation injury was not associated with physical, mental, or satisfaction with life outcomes at 24 months post-injury. This study adds to a limited number of studies examining the long-term outcomes and needs of individuals with inhalation injury.
